# Correlative Scanning Electron and Super-Resolution Structured Illumination Microscopy

**DOI:** 10.3390/photonics13080763

**Published:** 2026-08-14

**Authors:** Joseph R. Hamilton, Summer K. Levis, Guy M. Hagen

**Affiliations:** UCCS BioFrontiers Center, University of Colorado Colorado Springs, 1420 Austin Bluffs Parkway, Colorado Springs, CO 80918, USA

**Keywords:** structured illumination microscopy, scanning electron microscopy, surface shield enhancer, NanoSuit, correlative light and electron microscopy, super-resolution

## Abstract

Correlative microscopy techniques are used for many different applications in the biological sciences because the comparison of different imaging methods allows researchers to gain more and often complementary information about their samples. Correlative light and electron microscopy (CLEM) methods have been developed to preserve biological samples to withstand the harsh environments necessary for electron microscopy. After first being imaged using widefield (WF) and super-resolution structured illumination fluorescence microscopy (SIM), a NanoSuit chemical treatment was applied to a mammalian tissue sample before imaging with scanning electron microscopy (SEM). This was done to compare the image quality and resolution of each technique. SEM yields higher resolution and offers validation of results from SIM.

## 1. Introduction

Scanning electron microscopy (SEM) is a method used to obtain high-resolution images of microscopic samples, typically by coating the sample in a thin layer of conductive material and scanning the surface with an electron beam, generating an image. A high-vacuum environment is necessary for SEM but simultaneously creates undesirable conditions for biological samples by drying, dehydrating, or otherwise distorting them [1,2]. To combat these unfavorable outcomes, samples must be specially prepared prior to imaging by SEM. One such chemical treatment is the NanoSuit [3,4], which offers a layer of protection against the high-vacuum environment. A modification of the original NanoSuit method, called surface shield enhancer (SSE), consists of electrolytes and glycerol and protects the sample from moisture loss and other damage from the high-vacuum conditions and electron beam without compromising the quality and resolution of SEM imaging [5].

Although SEM provides information regarding the structure and surface of a sample, important functional details and insights into dynamics can only be obtained from other techniques, such as fluorescence microscopy [6]. However, optical microscopy is unable to attain the same high-resolution images that SEM does. The cooperative use of electron and optical microscopy—correlative light and electron microscopy (CLEM)—allows researchers to observe and relate structural and functional characteristics of biological samples by utilizing both SEM and optical microscopy on the same region of a biological sample [7]. The SSE NanoSuit is a useful chemical preparation for samples that undergo CLEM, as it provides the conductive and protective layer for SEM, preserves the quality of the sample, and can be removed to restore samples to their original state for further optical microscopy [8,9].

The choice of a specific optical microscopy technique is central to the success of CLEM in research applications. Fluorescence microscopy allows for 3D imaging of the inside of cells and tissues that have been labeled with fluorescent molecules to gain functional information about the sample but is hindered by the diffraction limit, thus generating low-resolution images. To combat this, structured illumination microscopy (SIM) [10-14], a fluorescence microscopy technique that uses shifting illumination patterns to generate a set of images, was designed to accomplish the acquisition of optically sectioned and/or super-resolution images. Maximum a posteriori (MAP)-SIM [15] is an evolution of standard SIM methods and is useful for imaging past the diffraction limit at increased sample depths [10]. To process the data, we used SIMToolbox [16], a MATLAB software version 2.0 that generates widefield (WF), optically sectioned SIM (OS-SIM) [14], and super-resolution MAP-SIM images from a single set of data.

These techniques were used to image a rat epididymis tissue sample with SEM, widefield fluorescence microscopy (WF), and MAP-SIM. The high-resolution structural data from SEM and the information derived from super-resolution fluorescence imaging via SIM can be combined to enhance the strengths of each technique and to obtain as much information as possible from a particular sample. This approach can potentially be applied to live biological samples [17-19] with use of the NanoSuit method and has applications in biomedical research, histology, pathology, and potentially numerous other research fields that utilize high-resolution imaging.

## 2. Materials and Methods

### 2.1. Surface Shield Enhancer (SSE) NanoSuit Preparation

To prepare the SSE solution we dissolved 5.0 g of sucrose, 5.0 g of fructose, 5.0 g of sodium chloride, 1.25 g of citric acid and 0.05 g of sodium glutamate in 500 mL ultra-pure water. To create a 1:2 ratio for the final solution, 150 mL SSE was combined with 300 mL glycerol [1]. The final pH was 7.4.

### 2.2. Slide Preparation

A few prepared slides were selected (Carolina Biological, Burlington, NC, USA, item 31-6464, rat epididymis, thickness ~ 15 μm). The tissue samples were stained by the manufacturer with hematoxylin and eosin (H&E) and are brightly fluorescent. In this type of staining, the fluorescence is due to the dye eosin (λ_max_ ex. 525 nm, λ_max_ em. 546 nm), which non-specifically stains protein within the tissue. H&E staining is usually highly reproducible but varies somewhat, depending on the quality of the prepared slides. The slides were imaged with SIM, as described below, and subsequently placed in toluene until the coverslip detached. The samples were then rehydrated in 100%, 90%, and 70% ethanol, respectively, for five minutes each, and finally in deionized water for a further five minutes. The sample slides were then dipped in the SSE/glycerol solution for 10 min at room temperature [8].

### 2.3. SEM Imaging

One of the sample slides was coated with gold for 100 sec. using a sputter coating machine (Desk V, Denton Vacuum, NJ, USA). This produced a layer of gold 4.3 nm thick as measured by an Inficon SQM-160 thickness monitor. Once the gold conductive layer was applied, the slide was cut (to reduce its size for the SEM chamber) with a diamond tip glass cutter, then placed into the SEM. The sample was then imaged at 30 kV (working distance 16 mm, chamber pressure approx. 1.4 × 10^−4^ Torr) using a Vega 3 SEM (Tescan, Brno, Czech Republic). We used the secondary electron detector and scan speed 5, resulting in an imaging time of about 2 min per SEM image.

### 2.4. SIM Imaging

We used a home-built SIM setup based on the same design as described previously [10,15]. The SIM system is based on a IX83 microscope (Olympus, Tokyo, Japan) equipped with a Zyla 4.2+ sCMOS camera (Andor, Belfast, UK) under the control of IQ software version 3.0 (Andor). For SIM imaging the following Olympus objectives were used: UPLSAPO 20×/0.85 NA oil immersion, WD 0.17 mm, and UPLSAPO 100×/1.4 NA oil immersion, WD 0.13 mm. The microscope is equipped with a filter set appropriate for TRITC (ET series, Chroma, Bellows Falls, VT, USA) and a motorized X, Y, piezo Z stage (Applied Scientific Instrumentation, Eugene, OR, USA). The camera and XYZ stage are controlled directly by Andor IQ software and an input/output computer card (DDA06/16, Measurement Computing, Norton, MA, USA; the card is controlled by Andor IQ software). Figure 1 shows a simplified diagram of the SIM system.

The SIM system uses a ferroelectric liquid crystal on silicon (LCOS) microdisplay (type SXGA-3DM, Forth Dimension Displays, Dalgety Bay, Fife, UK). This device has been used previously in SIM and fluorescence microscopy [15,16,20-26], allowing one to produce patterns of illumination on the sample, which can be manipulated by changing the image displayed on the microdisplay. This type of reflective microdisplay works by rotating the polarization of light by about 90 degrees when a pixel is in the ON state. In our setup, it is necessary to illuminate the microdisplay with vertically polarized light, which is reflected toward the LCOS by a polarizing beam splitter (PBS) cube (Thor Labs, Newton, NJ, USA). A pixel in the ON state will rotate the polarization to horizontal, which reflects off of the microdisplay and passes through the PBS cube and heads toward the back of the microscope. An 180 mm focal length lens (part SWTLU-C, Olympus) is attached to the back of the microscope and, together with the objective lens, relays an image of the microdisplay onto the sample. The microdisplay is operated using Metrocon software version 3.1 provided by the manufacturer (Forth Dimension Displays).

Figure 2A shows a photograph of the illumination pathway for the SIM system. The LCOS microdisplay is mounted on a XYZ translation stage (Thor Labs) for precise positioning. A linear polarizer is mounted in a rotation stage (both from Thor Labs) to allow adjustment for vertical polarization. Figure 2B shows a close-up of the LCOS microdisplay. Figure 2C shows another view of the LCOS illumination pathway, and Figure 2D shows the sample stage of the IX83 microscope.

The microdisplay used in our setup will not produce a SIM pattern on the sample if it is illuminated with a constant light source. The light source must be synchronized with an enable signal that is output from the microdisplay control board. Therefore, the wavelength selection signals output by the I/O card are first combined with the microdisplay enable signal. This is performed by the leftmost AND gates pictured in Figure 3. To reduce light exposure to the sample, the light source is turned off whenever the camera sensor is not being exposed. This is accomplished by performing a second logical AND of the result of the first AND with the ‘FIRE’ signal output from the camera. This is illustrated in Figure 3. The light source (Lumencor Spectra-X, Beaverton, OR, USA) is coupled to the system using a 1.5 mm diameter multi-mode optical fiber (Thor Labs). The Lumencor Spectra-X is a non-laser light source and produces incoherent illumination. This is important when considering the optical transfer function of the microscope, which for our setup is the completely incoherent case [27].

SIMToolbox software [16] was used to process the SIM data, which generated widefield, OS-SIM, and MAP-SIM images. SIMToolbox is an open-source, modular set of functions for MATLAB equipped with a user-friendly graphical interface designed for processing two-dimensional and three-dimensional data acquired by structured illumination microscopy. To evaluate the resolution of the acquired SIM images, we calculated the power spectral density [27,28]. This is a Fourier-based method, which allows an unbiased and consistent measurement of the resolution achieved in each image.

In the following experiments, we used a SIM pattern consisting of two ‘ON’ columns of microdisplay pixels and 10 columns of ‘OFF’ pixels, with a shift of one column between images and a single pattern direction (a single pattern angle). The time required to acquire the SIM data was about 5 min, with an additional 5 min required for data processing. Accurate spatial mapping between the microdisplay and camera was achieved by a calibration method as previously described [25] based on a standard method [29].

## 3. Results

### 3.1. Overview Imaging

To collect an overview image, we used an UPLXAPO 10×/0.40 NA objective and DP28 color camera (Olympus). A transmitted light image and a widefield fluorescence image of a rat testis sample stained with hematoxylin and eosin were acquired. This is shown in Figure 4 and offers a low magnification overview of the type of sample used in this study. The hematoxylin and eosin staining produces the familiar pink (protein staining by eosin) and purple (nuclear staining by hematoxylin) pattern seen in Figure 4A. The dye eosin is fluorescent, producing a bright foreground on a dark background in a fluorescence microscope as seen in Figure 4B.

### 3.2. Correlative SIM-SEM Imaging

A region of interest of a second rat testis sample was imaged with conventional widefield fluorescence microscopy, MAP-SIM, and SEM to observe the different methods side-by-side. This is shown in Figure 5. WF and SIM imaging occurred first, followed by NanoSuit-SSE treatment, gold sputtering, and SEM.

Figure 6 shows a comparison of WF and MAP-SIM images acquired with a 100×/1.4 NA oil immersion objective and SEM imaging after NanoSuit-SSE treatment and gold coating.

We next used ImageJ version 1.54g to calculate the fast Fourier transforms (FFTs) of the images in Figure 6. This is shown in Figure 7A-C.

One can estimate the cut off frequency, and therefore the resolution, by inspecting the Fourier transform of an image in which the pixel size is known accurately (Figure 7A-C). We then used SRMeasure Toolbox software version 1.0 [28] to accurately calculate the cut off frequency. This is shown in Figure 7D-F. To accomplish this, we calculate the circularly averaged power spectral density [27,28]. Another popular approach is calculation of the Fourier ring (or shell) correlation [30]. Table 1 shows resolution measurements for the images in Figure 6. Resolution measurements for Figures 4 and 5 are shown in Supplementary Tables S1 and S2.

## 4. Discussion

### 4.1. Structured Illumination Microscopy

It has been difficult to obtain “ground truth” images when considering super-resolution microscopy. This is an important topic because both structured illumination microscopy and single molecule localization microscopy (PALM/STORM type imaging [31-34]) rely on reconstruction algorithms to obtain the final image. These reconstruction algorithms are under constant development, and their reliability and possible potential to introduce artifacts are of concern [35]. Typically, conventional widefield images are shown in comparison to super-resolution images that were obtained by whichever method is being developed or applied. However, comparison of super-resolution microscopy images with SEM provides a higher-resolution image for comparison and helps prove that the super-resolution optical microscopy image is accurate and free from artifacts. The results shown here indicate that super-resolution structured illumination microscopy, as implemented by us (MAP-SIM), is showing the correct sample structure and is free from apparent artifacts. Other SIM methods are more prone to pattern-related artifacts [36].

In our setup, we used a liquid crystal on silicon (LCOS) microdisplay. The wavelength range of the LCOS microdisplay is 430 nm to 700 nm, as given by the manufacturer (Forth Dimension Displays). Many other SIM systems [37] (and similar microscopy setups [38]) have instead used digital micromirror devices (DMDs). DMDs (also called digital light processing or DLP microdisplays) are produced by Texas Instruments (Dallas, TX, USA). DLP microdisplays are available in several different wavelength ranges including UV (355–420 nm), visible (400–700 nm) and near-IR (700–2500 nm). So, for visible light operation, the two types of microdisplay (LCOS and DLP) are quite comparable. However, 405 nm is an important wavelength in optical microscopy (for example, when using photoconvertible fluorescent proteins [39], so the DLP would be a better choice. On the other hand, DLPs are more difficult to integrate into an optical system. This is because the regular array of tilted mirrors used in DLP microdisplays act as diffraction grating, causing light to be reflected into several diffraction orders [40]. Additionally, DLP microdisplays must be “fed on the diagonal”, meaning the incoming light must be directed onto the display at a diagonal angle tilted away from the optical axis, leading to a three-dimensional arrangement.

### 4.2. Correlative Light and Electron Microscopy

Other correlative microscopy techniques differ in procedure but serve a similar function in obtaining information about a sample (including non-biological materials [41]) from complementary methods [42,43]. Another type of CLEM involves the use of widefield or laser scanning confocal microscopy (LSCM) followed by transmission electron microscopy (TEM) [44-47]. The images captured from TEM and LSCM can be overlaid into an integrated image [48], providing, for example, ultrastructural imaging of cells rapidly frozen at a moment in time at which a particular cellular function is taking place. When creating such an overlay image, different image quality and properties between the two images can make this task difficult, leading to innovative algorithms for this purpose [49]. An example of a side-by-side integrated image is shown in Supplementary Figure S1.

An ongoing challenge is refinement of labeling strategies for CLEM [45]. A recent (2026) approach was able to visualize lipid distribution in cells using CLEM with lipid probes and on-section click chemistry methods [50], while another method used a novel red fluorescent protein for labeling of several different organelles [51].

## 5. Conclusions

CLEM methods have been used in many diverse areas of bio-research including studies of plants [52,53], neuroscience [54-56], mitochondria [57], and many other areas [42]. Procedures for correlative microscopy techniques have been in development for many years and are constantly being modified to maximize the benefits from their use. The utilization of super-resolution SIM with SEM is an especially promising form of CLEM due to the relative ease of the method and surprisingly good results, as shown here.

## Supplementary Material

Supplement

**Supplementary Materials:** The following supporting information can be downloaded at: https://www.mdpi.com/article/10.3390/photonics13080763/s1, Figure S1: Optional creation of a side-by-side presentation. Table S1: Resolution measurements for Figure 4 (overview at 10×). Table S2: Resolution measurements for Figure 5 (correlative images at 20×).

## Figures and Tables

**Figure 1.**
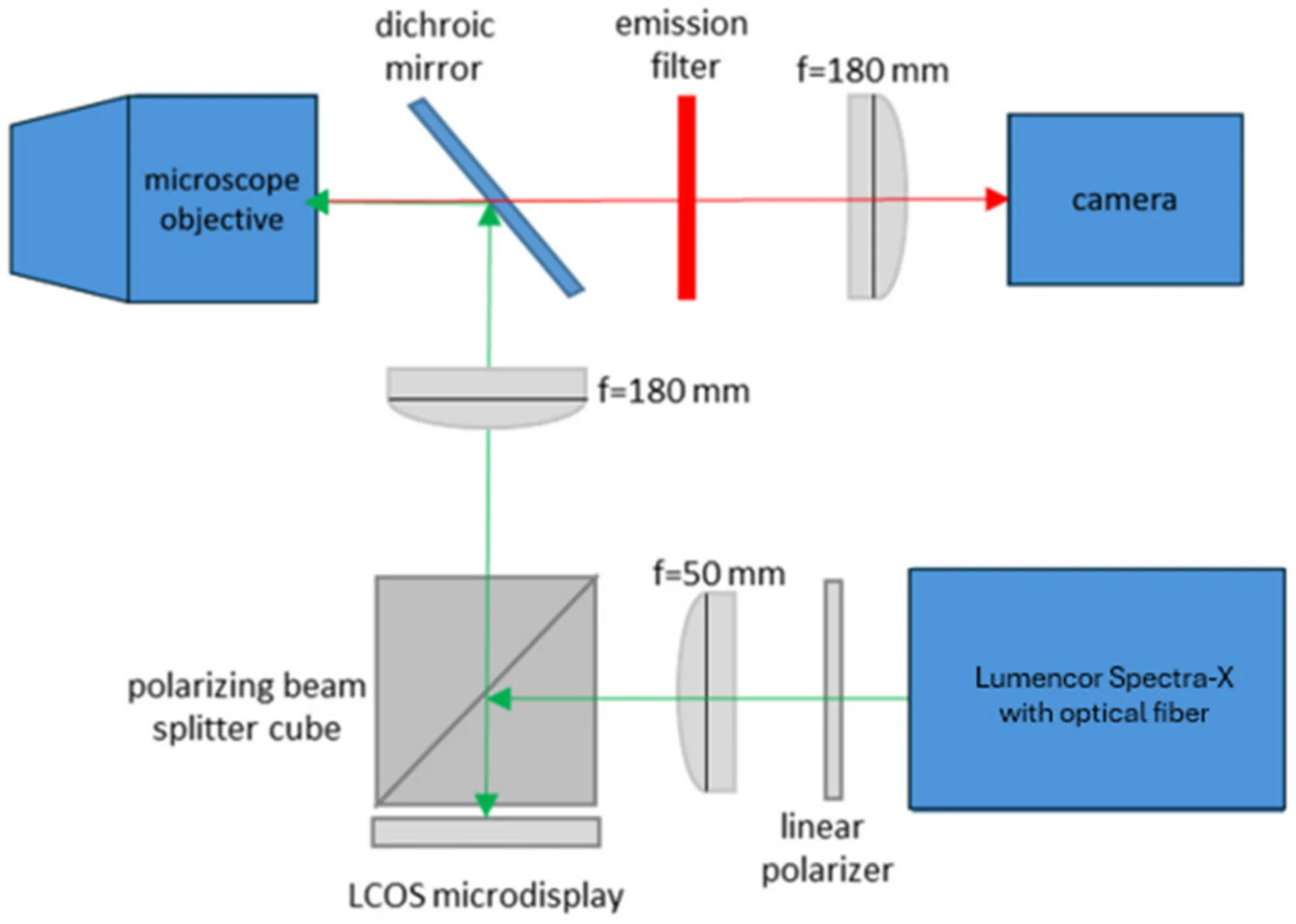
Simplified diagram of SIM system. LCOS, liquid crystal on silicon.

**Figure 2.**
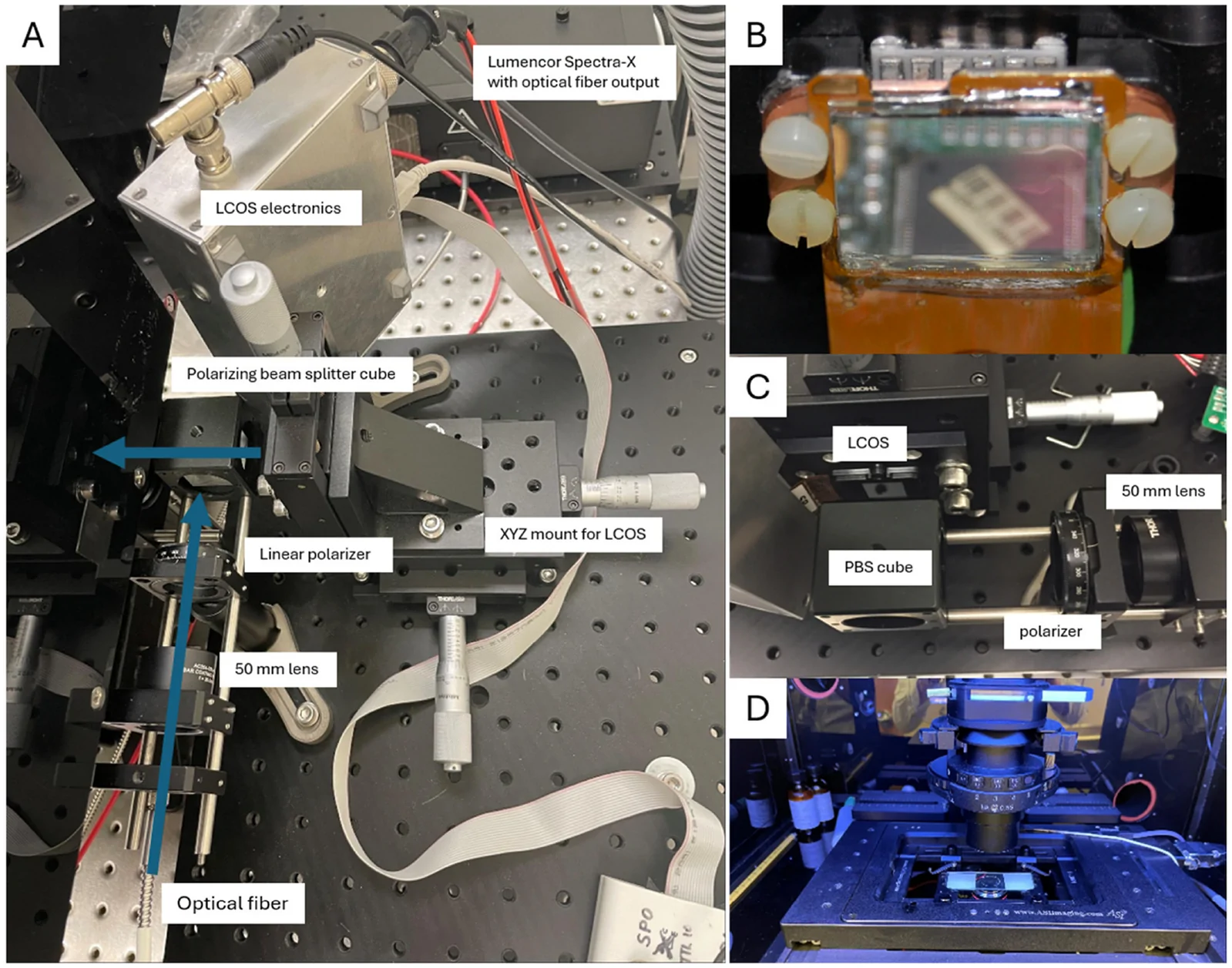
(**A**) Photograph of the SIM illumination pathway. (**B**) Close-up of the LCOS microdisplay. (**C**) View of the SIM illumination pathway. (**D**) Sample stage of the IX83 microscope with an incubator chamber.

**Figure 3.**
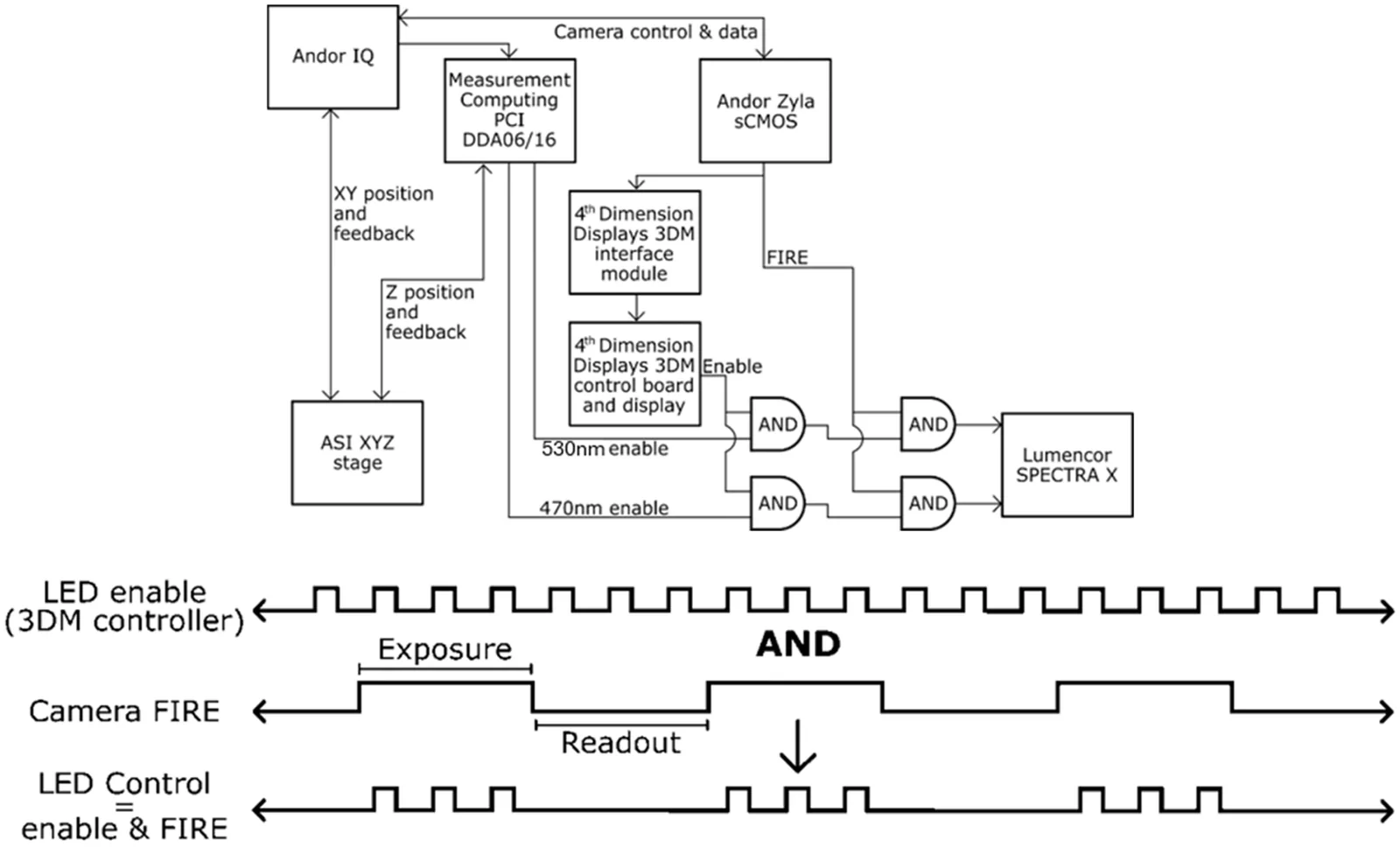
SIM system details. (Above) Connection diagram; (below) schematic of the timing scheme. The diagram shows the operation for two wavelengths (470 nm and 530 nm). In this study, we used only a single wavelength for excitation (530 nm).

**Figure 4.**
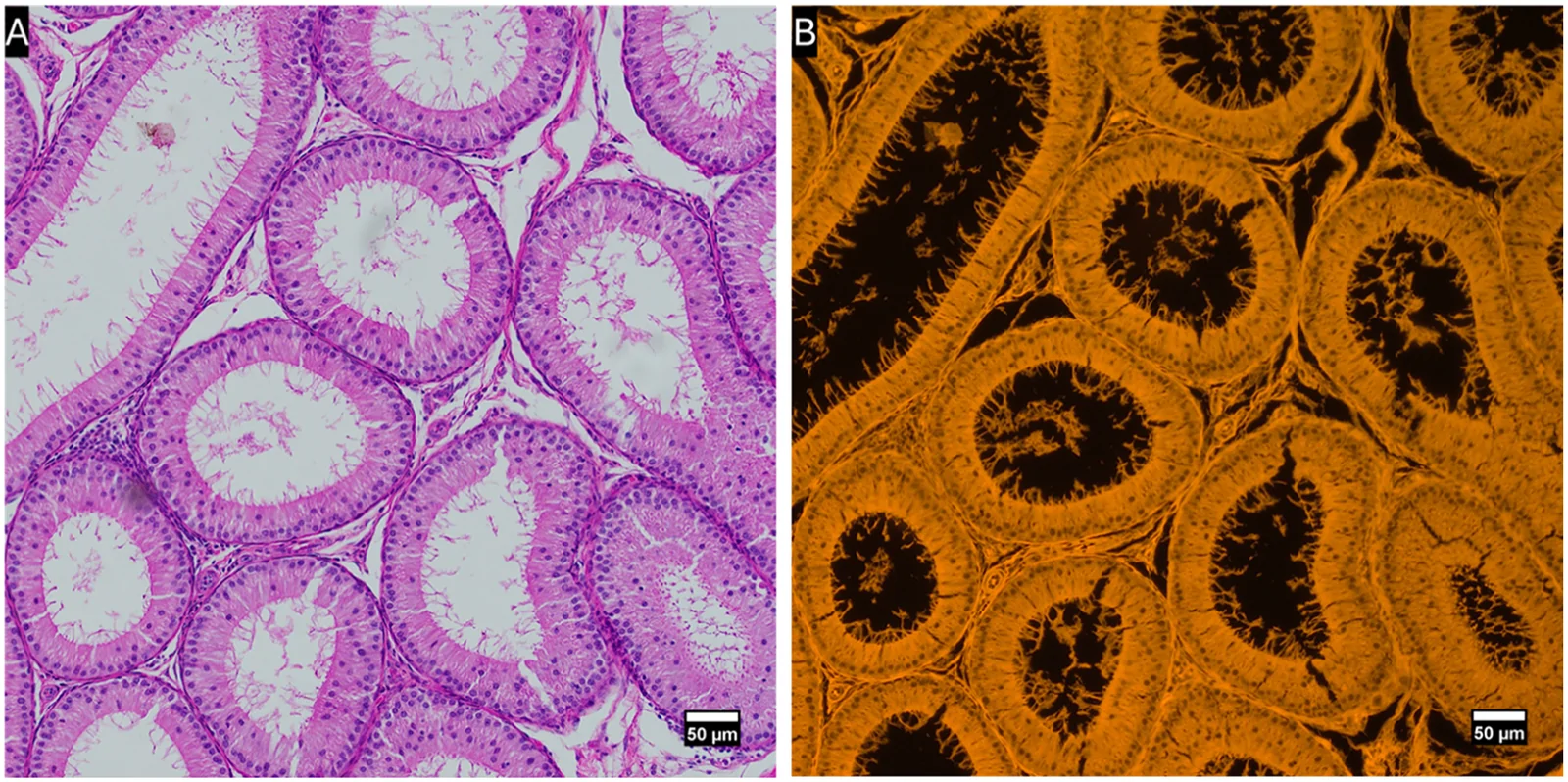
(**A**) Representative transmitted light and (**B**) fluorescence images of a rat testis sample using a UPLXAPO 10×/0.4 NA objective and DP28 color camera.

**Figure 5.**
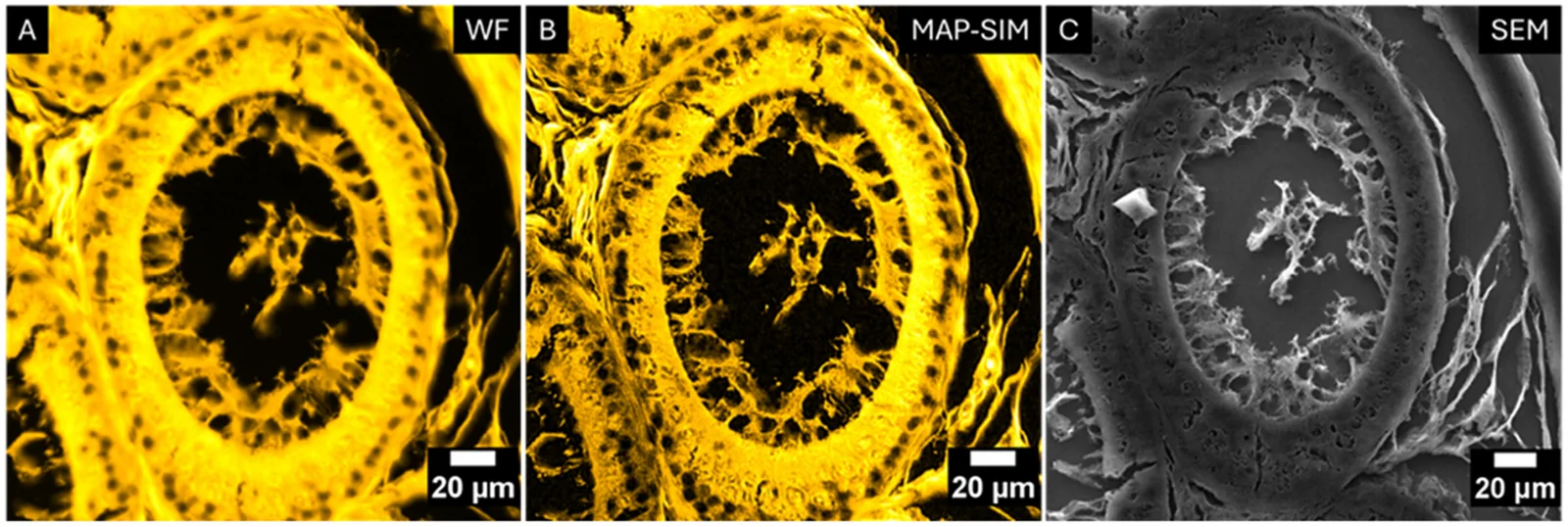
Correlative fluorescence and scanning electron microscopy of rat testis tissue stained with H&E. (**A**) Conventional WF; (**B**) MAP-SIM; 20×/0.85 NA objective. (**C**) SEM after treatment with SSE and gold coating.

**Figure 6.**
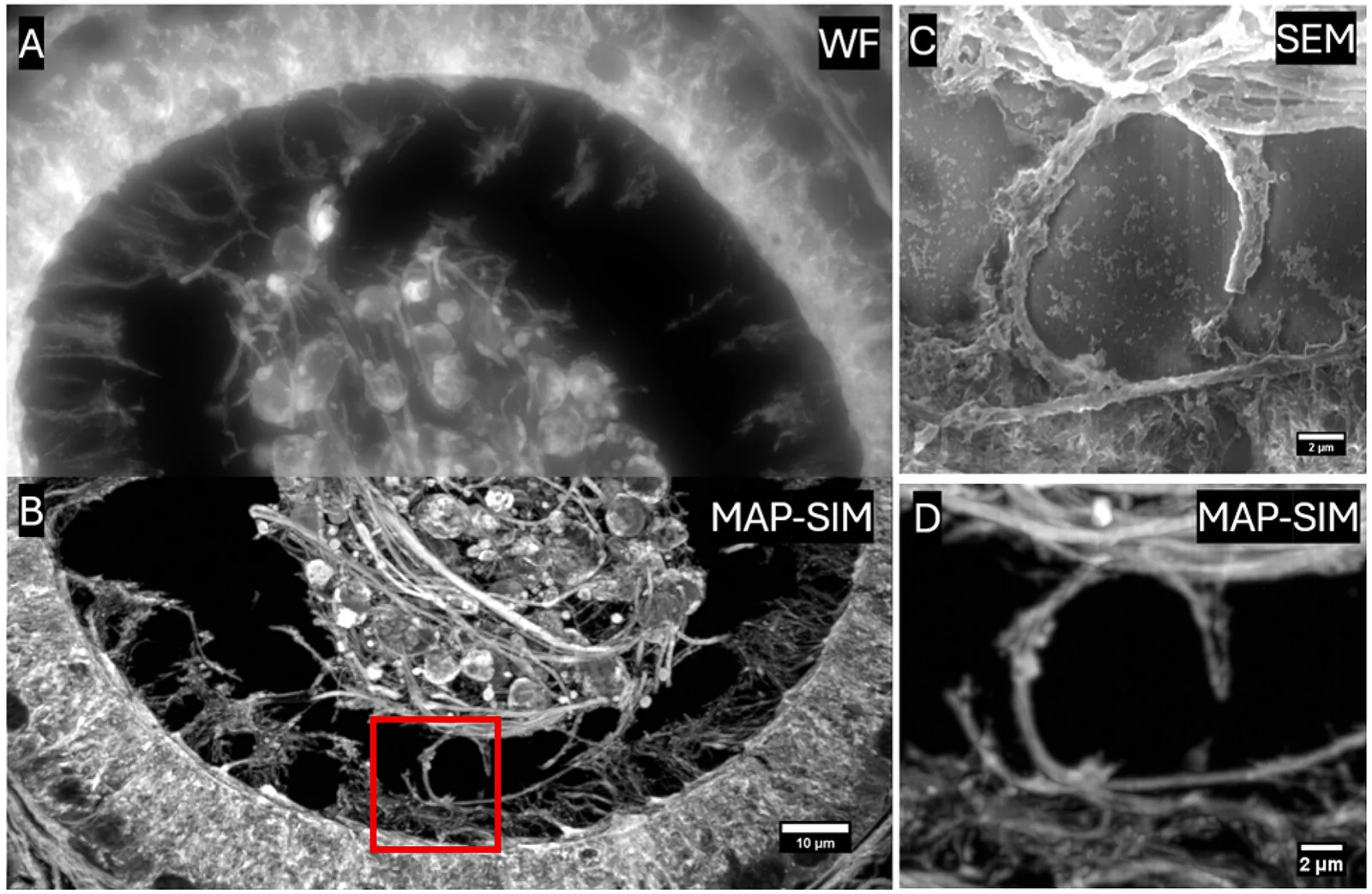
Correlative widefield, super-resolution structured illumination, and scanning electron microscopy. (**A**) WF, (**B**) MAP-SIM (both 100×/1.4 NA). (**C**) SEM, with region of interest indicated by the red box in panel B. (**D**) MAP-SIM of the same region of interest.

**Figure 7.**
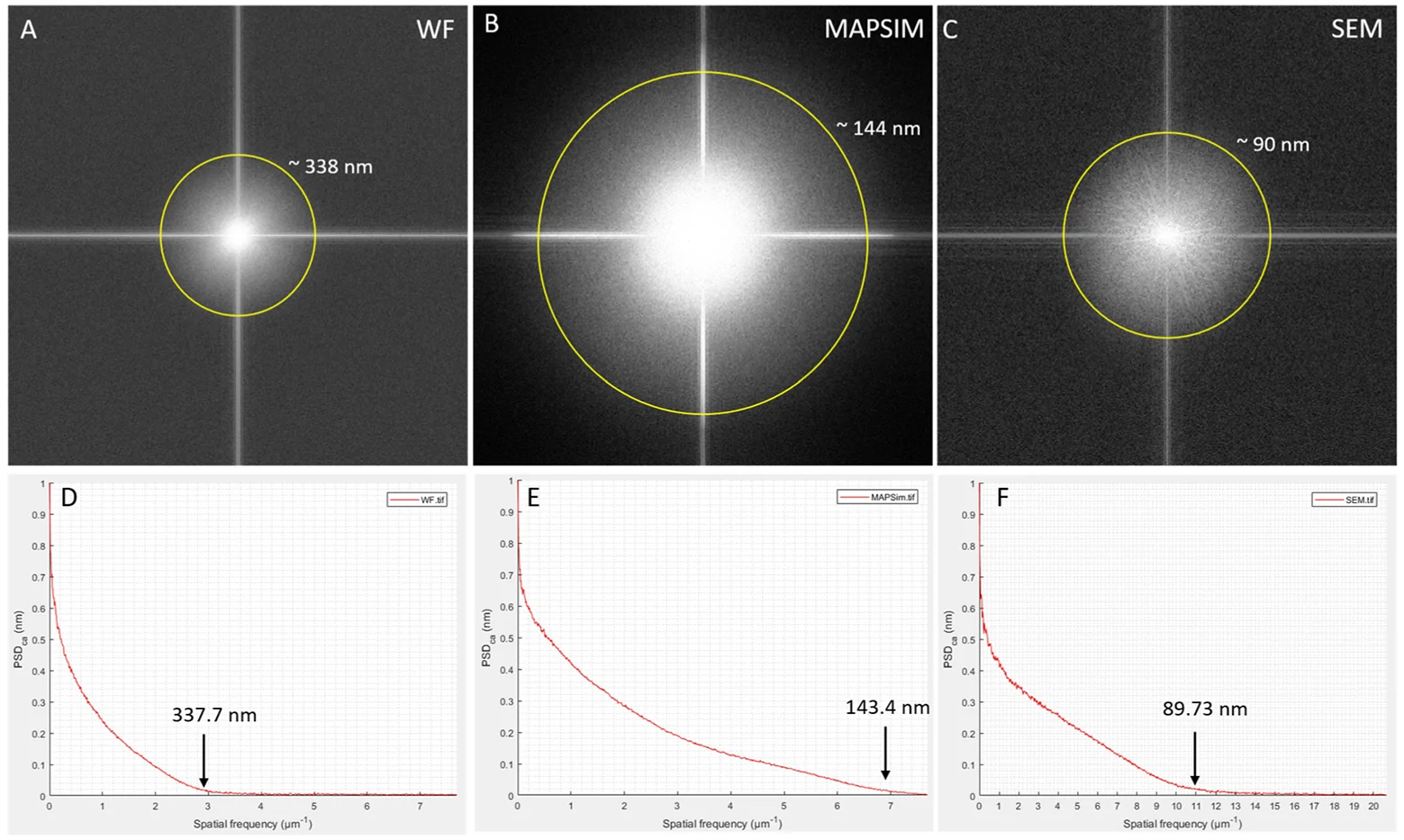
FFTs of images in Figure 6. (**A**) WF, (**B**) MAP-SIM, (**C**) SEM. (**D**) Power spectral density (PSD) plot of (**A**). (**E**) PSD plot of (**B**). (**F**) PSD plot of (**C**).

**Table 1.** Resolution measurements of the images in Figure 6.

	WF	SIM	SEM
Image pixel size	65 nm	65 nm	24.185 nm
Resolution measurement	337.7 nm	143.4 nm	89.73 nm
Electron beam spot size	N/A	N/A	87.6 nm

## Data Availability

Data are available upon request.

## Abbreviations

The following abbreviations are used in this manuscript:

H&E: Hematoxylin and eosin
MAP-SIM: Maximum a posteriori probability
SSE: Surface shield enhancer
NA: Numerical aperture
LCOS: Liquid crystal on silicon
CLEM: Correlative light and electron microscopy
SEM: Scanning electron microscopy
SIM: Structured illumination microscopy
WD: Working distance

## References

[1] Takaku Y; Suzuki H; Ohta I; Tsutsui T; Matsumoto H; Shimomura M; Hariyama T. A ‘NanoSuit’ Surface Shield Successfully Protects Organisms in High Vacuum: Observations on Living Organisms in an FE-SEM. Proc. R. Soc. B Biol. Sci. 2015, 282, 20142857. 10.1098/rspb.2014.2857 PMC4344158 25631998

[2] Ohta I; Takaku Y; Suzuki H; Ishii D; Muranaka Y; Shimomura M; Hariyama T. Dressing Living Organisms in a Thin Polymer Membrane, the Nanosuit, for High-Vacuum FE-SEM Observation. Microscopy 2014, 63, 295–300. 10.1093/jmicro/dfu015 24824083

[3] Takaku Y; Suzuki H; Ohta I; Ishii D; Muranaka Y; Shimomura M; Hariyama T. A Thin Polymer Membrane, Nano-Suit, Enhancing Survival across the Continuum between Air and High Vacuum. Proc. Natl. Acad. Sci. USA 2013, 110, 7631–7635. 10.1073/pnas.1221341110 PMC3651493 23589878

[4] Takaku Y. NanoSuit Method for the Observation of Living/Hydrous Organisms in an SEM. Microsc. Microanal. 2022, 28, 1388.

[5] Takaku Y; Suzuki H; Kawasaki H; Ohta I; Ishii D; Hirakawa S; Tsutsui T; Matsumoto H; Takehara S; Nakane C; A Modified ‘NanoSuit’ Preserves Wet Samples in High Vacuum: Direct Observations on Cells and Tissues in Field-Emission Scanning Electron Microscopy. R. Soc. Open Sci. 2017, 4, 160887. 10.1098/rsos.160887 PMC5383832 28405375

[6] Liv N; Zonnevylle AC; Narvaez AC; Effting APJ; Voorneveld PW; Lucas MS; Hardwick JC; Wepf RA; Kruit P; Hoogenboom JP. Simultaneous Correlative Scanning Electron and High-NA Fluorescence Microscopy. PLoS ONE 2013, 8, e55707. 10.1371/journal.pone.0055707 PMC3568124 23409024

[7] Begemann I; Viplav A; Rasch C; Galic M. Stochastic Micro-Pattern for Automated Correlative Fluorescence—Scanning Electron Microscopy. Sci. Rep. 2015, 5, 17973. 10.1038/srep17973 PMC4673610 26647824

[8] Kawasaki H; Itoh T; Takaku Y; Suzuki H; Kosugi I; Meguro S; Iwashita T; Hariyama T. The NanoSuit Method: A Novel Histological Approach for Examining Paraffin Sections in a Nondestructive Manner by Correlative Light and Electron Microscopy. Lab. Investig. 2020, 100, 161–173. 10.1038/s41374-019-0309-7 PMC6917571 31467424

[9] Shinmura K; Kawasaki H; Baba S; Ohta I; Kato H; Yasuda H; Yamada S; Misawa K; Sugimoto K; Osawa S; Utility of Scanning Electron Microscopy Elemental Analysis Using the ‘Nanosuit’ Correlative Light and Electron Microscopy Method in the Diagnosis of Lanthanum Phosphate Deposition in the Esophagogastroduodenal Mucosa. Diagnostics 2020, 10, 1. 10.3390/diagnostics10010001 PMC7167806 31861386

[10] Paul TC; Johnson KA; Hagen GM. Super-Resolution Imaging of Neuronal Structures with Structured Illumination Microscopy. Bioengineering 2023, 10, 1081. 10.3390/bioengineering10091081 PMC10525192 37760183

[11] Johnson KA; Hagen GM. Artifact-Free Whole-Slide Imaging with Structured Illumination Microscopy and Bayesian Image Reconstruction. Gigascience 2021, 9, giaa035. 10.1093/gigascience/giaa035 PMC7155289 32285910

[12] Gustafsson MGL. Surpassing the Lateral Resolution Limit by a Factor of Two Using Structured Illumination Microscopy. J. Microsc. 2000, 198, 82–87. 10.1046/j.1365-2818.2000.00710.x 10810003

[13] Heintzmann R; Cremer CG. Laterally Modulated Excitation Microscopy: Improvement of Resolution by Using a Diffraction Grating. In Proceedings of the Optical Biopsies and Microscopic Techniques III; SPIE: Bellingham, WA, USA, 1999; Volume 3568, pp. 185–196.

[14] Neil MAA; Juškaitis R; Wilson T. Method of Obtaining Optical Sectioning by Using Structured Light in a Conventional Microscope. Opt. Lett. 1997, 22, 1905. 10.1364/ol.22.001905 18188403

[15] Lukeš T; Křížek P; Švindrych Z; Benda J; Ovesný M; Fliegel K; Klíma M; Hagen GM. Three-Dimensional Super-Resolution Structured Illumination Microscopy with Maximum a Posteriori Probability Image Estimation. Opt. Express 2014, 22, 29805–29817. 10.1364/OE.22.029805 25606910

[16] Křížek P; Lukeš T; Ovesný M; Fliegel K; Hagen GM. SIMToolbox: A MATLAB Toolbox for Structured Illumination Fluorescence Microscopy. Bioinformatics 2015, 32, 318–320. 10.1093/bioinformatics/btv576 26446133

[17] Takehara S; Takaku Y; Suzuki H; Ohta I; Shimomura M; Hariyama T A “NanoSuit” Successfully Protects Petals of Cherry Blossoms in High Vacuum: Examination of Living Plants in an FE-SEM. Sci. Rep. 2018, 8, 1685. 10.1038/s41598-018-19968-w PMC5785992 29374227

[18] Fujita M; Blanke A; Nomura S; Machida R. Simple, Artifact-Free SEM Observations of Insect Embryos: Application of the Nano-Suit Method to Insect Embryology. Proc. Arthropod. Embryol. Soc. Jpn. 2016, 50, 7–10.

[19] Takaku Y; Takehara S; Suzuki C; Suzuki H; Shimomura M; Hariyama T. In Situ Elemental Analyses of Living Biological Specimens Using ‘NanoSuit’ and EDS Methods in FE-SEM. Sci. Rep. 2020, 10, 14574. 10.1038/s41598-020-71523-8 PMC7471950 32884008

[20] Hagen GM; Caarls W; Lidke KA; De Vries AHB; Fritsch C; Barisas BG; Arndt-Jovin DJ; Jovin TM. Fluorescence Recovery after Photobleaching and Photoconversion in Multiple Arbitrary Regions of Interest Using a Programmable Array Microscope. Microsc. Res. Tech. 2009, 72, 431–440. 10.1002/jemt.20686 PMC3131617 19208387

[21] Shao L; Kner P; Rego EH; Gustafsson MGL. Super-Resolution 3D Microscopy of Live Whole Cells Using Structured Illumination. Nat. Methods 2011, 8, 1044–1048. 10.1038/nmeth.1734 22002026

[22] Fiolka R; Shao L; Rego EH; Davidson MW; Gustafsson MGL. Time-Lapse Two-Color 3D Imaging of Live Cells with Doubled Resolution Using Structured Illumination. Proc. Natl. Acad. Sci. USA 2012, 109, 5311–5315. 10.1073/pnas.1119262109 PMC3325651 22431626

[23] Chen BC; Legant WR; Wang K; Shao L; Milkie DE; Davidson MW; Janetopoulos C; Wu XS; Hammer JA; Liu Z; Lattice Light-Sheet Microscopy: Imaging Molecules to Embryos at High Spatiotemporal Resolution. Science 2014, 346, 1257998. 10.1126/science.1257998 PMC4336192 25342811

[24] Hagen GM; Caarls W; Thomas M; Hill A; Lidke KA; Rieger B; Fritsch C; van Geest B; Jovin TM; Arndt-Jovin DJ. Biological Applications of an LCoS-Based Programmable Array Microscope (PAM). Imaging Manip. Anal. Biomol. Cells Tissues V 2007, 6441, 218–229.

[25] Křížek P; Raška I; Hagen GM. Flexible Structured Illumination Microscope with a Programmable Illumination Array. Opt. Express 2012, 20, 24585. 10.1364/OE.20.024585 23187221

[26] Hannebelle MTM; Raeth E; Leitao SM; Lukeš T; Pospíšil J; Toniolo C; Venzin OF; Chrisnandy A; Swain PP; Ronceray N; Open-Source Microscope Add-on for Structured Illumination Microscopy. Nat. Commun. 2024, 15, 1550. 10.1038/s41467-024-45567-7 PMC10879112 38378733

[27] Goodman JW. Frequency Analysis of Optical Imaging Systems. In Introduction to Fourier Optics; McGraw-HIll Int.: New York, NY, USA, 1968; pp. 126–171.

[28] Pospíšil J; Fliegel K; Klíma M. Assessing Resolution in Live Cell Structured Illumination Microscopy. In Proceedings of SPIE—The International Society for Optical Engineering; Páta P, Fliegel K, Eds.; SPIE: Bellingham, WA, USA, 2017; Volume 10603, p. 39.

[29] Šonka M; Hlaváč V; Boyle R. Image Processing Analysis and Machine Vision, 2nd ed.; PWS Publishing: Boston, MA, USA, 1998.

[30] Nieuwenhuizen RPJ; Lidke KA; Bates M; Puig DL; Grünwald D; Stallinga S; Rieger B. Measuring Image Resolution in Optical Nanoscopy. Nat. Methods 2013, 10, 557–562. 10.1038/nmeth.2448 PMC4149789 23624665

[31] Ovesný M; Křížek P; Borkovec J; Švindrych Z; Hagen GM. ThunderSTORM: A Comprehensive ImageJ Plug-in for PALM and STORM Data Analysis and Super-Resolution Imaging. Bioinformatics 2014, 30, 2389–2390. 10.1093/bioinformatics/btu202 PMC4207427 24771516

[32] Shtengel G; Wang Y; Zhang Z; Goh WI; Hess HF; Kanchanawong P. Imaging Cellular Ultrastructure by PALM, IPALM, and Correlative IPALM-EM. Methods Cell Biol. 2014, 123, 273–294. 10.1016/B978-0-12-420138-5.00015-X 24974033

[33] Rust MJ; Bates M; Zhuang X. Sub-Diffraction-Limit Imaging by Stochastic Optical Reconstruction Microscopy (STORM). Nat. Methods 2006, 3, 793–795. 10.1038/nmeth929 PMC2700296 16896339

[34] Betzig E; Patterson GH; Sougrat R; Lindwasser OW; Olenych S; Bonifacino JS; Davidson MW; Lippincott-Schwartz J; Hess HF. Imaging Intracellular Fluorescent Proteins at Nanometer Resolution. Science 2006, 313, 1642–1645. 10.1126/science.1127344 16902090

[35] Chen X; Zhong S; Hou Y; Cao R; Wang W; Li D; Dai Q; Kim D; Xi P. Superresolution Structured Illumination Microscopy Reconstruction Algorithms: A Review. Light Sci. Appl. 2023, 12, 172. 10.1038/s41377-023-01204-4 PMC10336069 37433801

[36] Pospíšil J; Fliegel K; Klíma M. Analysis of Image Reconstruction Artifacts in Structured Illumination Microscopy. In Proceedings of the Applications of Digital Image Processing XL; Tescher AG, Ed.; SPIE: Bellingham, WA, USA, 2017; Volume 10396, pp. 1–12.

[37] Dan D; Lei M; Yao B; Wang W; Winterhalder M; Zumbusch A; Qi Y; Xia L; Yan S; Yang Y; DMD-Based LED-Illumination Super-Resolution and Optical Sectioning Microscopy. Sci. Rep. 2013, 3, 1116. 10.1038/srep01116 PMC3552285 23346373

[38] Hanley QS; Verveer PJ; Gemkow M; Arndt-Jovin DJ; Jovin TM. An Optical Sectioning Programmable Array Microscope Implemented with a Digital Micromirror Device. J. Microsc. 1999, 196, 317–331. 10.1046/j.1365-2818.1999.00602.x 10594772

[39] Hirvonen L; Mandula O; Wicker K; Heintzmann R. Structured Illumination Microscopy Using Photoswitchable Fluorescent Proteins. In Proceedings of the Three-Dimensional and Multidimensional Microscopy: Image Acquisition and Processing XV; Conchello J-A, Cogswell CJ, Wilson T, Eds.; SPIE: San Jose, CA, USA, 2008; Volume 6861, pp. 68610L.1–68610L.8.

[40] Brown PT; Kruithoff R; Seedorf GJ; Shepherd DP. Multicolor Structured Illumination Microscopy and Quantitative Control of Polychromatic Light with a Digital Micromirror Device. Biomed. Opt. Express 2021, 12, 3700. 10.1364/BOE.422703 PMC8221958 34221689

[41] Hutiu G; Duma VF; Demian D; Bradu A; Podoleanu AG. Assessment of Ductile, Brittle, and Fatigue Fractures of Metals Using Optical Coherence Tomography. Metals 2018, 8, 117.

[42] Ando T; Bhamidimarri SP; Brending N; Colin-York H; Collinson L; De Jonge N; De Pablo PJ; Debroye E; Eggeling C; Franck C; The 2018 Correlative Microscopy Techniques Roadmap. J. Phys. D. Appl. Phys. 2018, 51, 443001. 10.1088/1361-6463/aad055 PMC6372154 30799880

[43] Jeong D; Kim D. Recent Developments in Correlative Super-Resolution Fluorescence Microscopy and Electron Microscopy. Mol. Cells 2022, 45, 41–50. 10.14348/molcells.2021.5011 PMC8819494 35114646

[44] Verkade P; Kingdom U. Moving EM: The Rapid Transfer System as a New Tool for Correlative Light and Electron Microscopy and High Throughput for High-Pressure Freezing. J. Microsc. 2008, 230, 317–328. 10.1111/j.1365-2818.2008.01989.x 18445162

[45] Tanner H; Sherwin O; Verkade P. Labelling Strategies for Correlative Light Electron Microscopy. Microsc. Res. Tech. 2023, 86, 901–910. 10.1002/jemt.24304 36846978

[46] Erdmann M; Hodgson L; Webb I; Davidson AD; Verkade P. Severe Acute Respiratory Syndrome Coronavirus 2 (SARS-CoV2) Culture and Sample Preparation for Correlative Light Electron Microscopy. In Methods in Cell Biology; Academic Press: Cambridge, MA, USA, 2024; Volume 187, pp. 99–116. 10.1016/bs.mcb.2024.02.031 38705632

[47] Rieder CL; Bowser SS. Correlative Immunofluorescence and Electron Microscopy on the Same Section of Epon-Embedded Material. J. Histochem. Cytochem. 1985, 33, 165–171. 10.1177/33.2.3881520 3881520

[48] Keene DR; Tufa SF; Wong MH; Smith NR; Sakai LY; Horton WA. Correlation of the Same Fields Imaged in the TEM, Confocal, LM, and MicroCT by Image Registration: From Specimen Preparation to Displaying a Final Composite Image. Methods Cell Biol. 2014, 124, 391–417. 10.1016/B978-0-12-801075-4.00018-5 25287851

[49] Krentzel D; Elphick M; Domart MC; Peddie CJ; Laine RF; Shand C; Henriques R; Collinson LM; Jones ML. CLEM-Reg: An Automated Point Cloud-Based Registration Algorithm for Volume Correlative Light and Electron Microscopy. Nat. Methods 2025, 22, 1923–1934. 10.1038/s41592-025-02794-0 PMC12446066 40931193

[50] Lennartz HM; Khan S; Leng W; Böhlig K; Fabig G; Kieswald Y; Elsner F; Scher N; Wilsch-Bräuninger M; Avinoam O; Visualizing Suborganellar Lipid Distribution Using Correlative Light and Electron Microscopy. Nat. Cell Biol. 2026, 28, 828–837. 10.1038/s41556-026-01915-x PMC13086582 41862633

[51] Xiong H; Chang Q; Ding J; Wang S; Zhang W; Li Y; Wu Y; Lin P; Yang C; Liu M; A Highly Stable Monomeric Red Fluorescent Protein for Advanced Microscopy. Nat. Methods 2025, 22, 1288–1298. 10.1038/s41592-025-02676-5 40247125

[52] Franek M; Koptašíková L; Mikšátko J; Liebl D; Macíčková E; Pospíšil J; Esner M; Dvořáčková M; Fajkus J. In-Section Click-IT Detection and Super-Resolution CLEM Analysis of Nucleolar Ultrastructure and Replication in Plants. Nat. Commun. 2024, 15, 2445. 10.1038/s41467-024-46324-6 PMC10950858 38503728

[53] Daraspe J; Bellani E; De Bellis D; Genoud C; Geldner N. Targeted Imaging of Specialized Plant Cell Walls by Improved Cryo-CLEM and Cryo-Electron Tomography. Nat. Commun. 2025, 16, 11354. 10.1038/s41467-025-66410-7 PMC12728224 41387421

[54] Modla S; Mendonca J; Czymmek KJ; Akins RE. Identification of Neuromuscular Junctions by Correlative Confocal and Transmission Electron Microscopy. J. Neurosci. Methods 2010, 191, 158–165. 10.1016/j.jneumeth.2010.06.015 PMC3144377 20600319

[55] Shafiei N; Stähli D; Burger D; Di Fabrizio M; van den Heuvel L; Daraspe J; Böing C; Shahmoradian SH; van de Berg WDJ; Genoud C; Correlative Light and Electron Microscopy for Human Brain and Other Biological Models. Nat. Protoc. 2025, 20, 2994–3023. 10.1038/s41596-025-01153-9 40164750

[56] Wurm CA; Schwarz H; Jans DC; Riedel D; Humbel BM; Jakobs S. Correlative STED Super-Resolution Light and Electron Microscopy on Resin Sections. J. Phys. D. Appl. Phys. 2019, 52, 374003.

[57] Kopek BG; Shtengel G; Xu CS; Clayton DA; Hess HF. Correlative 3D Superresolution Fluorescence and Electron Microscopy Reveal the Relationship of Mitochondrial Nucleoids to Membranes. Proc. Natl. Acad. Sci. USA 2012, 109, 6136–6141. 10.1073/pnas.1121558109 PMC3341004 22474357

