# Supplementary material for: Correlative Scanning Electron and Super-Resolution Structured Illumination Microscopy
Source: Photonics. Author manuscript; Available in PMC 2026 Nov 6. (PMC13739454; doi:10.3390/photonics13080763)
Supplement: Supplementary file 1 [file NIHMS2220116-supplement-Supplement.pdf]

Supplementary Materials

Figure S1 shows an optional step, creating an overlapping correlative image. This step is not always necessary, but it does offer a side-by-side comparison of the two imaging methods. One possible process consists of: collection of (A) SEM and (B) fluorescence images, (C,D) cropping, (E) manual resizing and X,Y positioning of the fluorescence image on top of the SEM image, and (F) final side-by-side presentation.

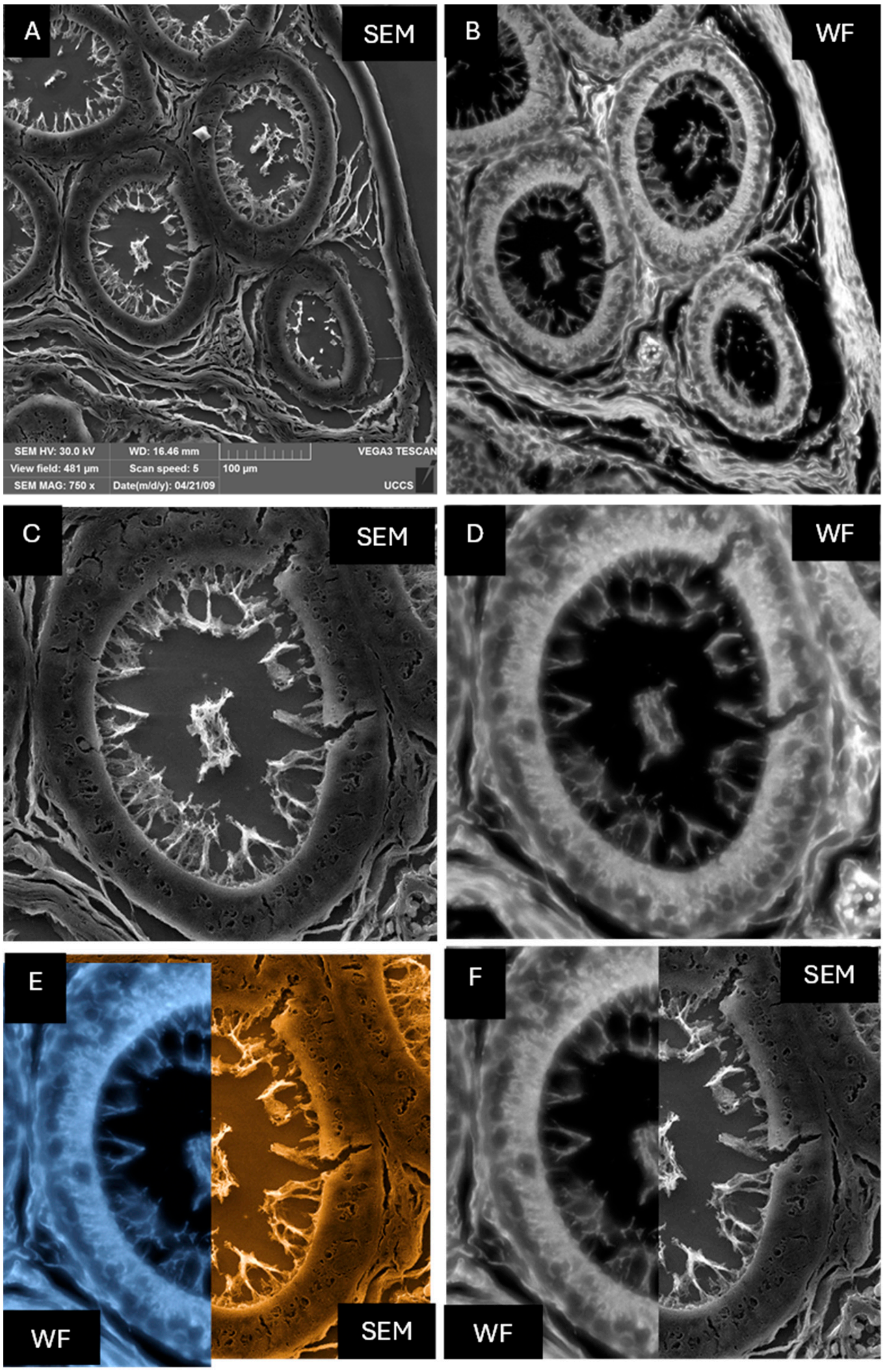

Figure S1: Optional creation of a side-by-side presentation. (A) SEM, (B) WF fluorescence, (C) SEM region of interest, (D) WF region of interest, (E) overlap image after scaling and XY positioning, (F)

final side-by-side image allowing direct comparison of fluorescence and electron microscope results.

Resolution measurements for Figures 4 and 5:

Table S1: Resolution measurements for Figure 4 (overview at 10×)

	**Fluorescence**	**Transmitted light**
Image pixel size	345 nm	345 nm
Resolution measurement	743 nm	794.8 nm

Table S2: Resolution measurements for Figure 5 (correlative images at 20×)

	**WF**	**SIM**	**SEM**
Image pixel size	325 nm	325 nm	376 nm
Resolution measurement	1,058 nm	704.8 nm	925 nm
Electron beam spot size	N/A	N/A	92.5 nm
